# Supplementary material for: Cytokine Profiling of Amniotic Fluid from Congenital Cytomegalovirus Infection
Source: Viruses. 2022 Sep 28;14(10):2145. doi: 10.3390/v14102145 (PMC9607316; doi:10.3390/v14102145)

Table S1. Summary of neonatal assessment of infected neonates.

SampleID	Fetal gender	GA amniocentesis	VL amniotic fluid (log)	GA cordocentesis	VL fetal blood	Fetal platele	Prenatal imaging	Pregancy outcome	BW <3rd percentile	TF-US	AABR	FE	Symptomatic asymptomatic newborn
7	1	23.57	7	24.57	3.9	139000	HEB	LB	no	normal	normal	normal	asymptomatic
25	1	22.29	6.2	23.86	3	196000	normal	LB	no	normal	normal	normal	asymptomatic
27	1	20.71	6.3	23	4.4	195000	normal	LB	no	normal	normal	normal	asymptomatic
35	2	20.29	7.1	22	6.3	171000	PIMG, SMG, HEB	LB	no	normal	normal	normal	asymptomatic
37	1	21.57	6.1	22.43	5	132000	SMG, HMG, HEB	LB	no	normal	normal	normal	asymptomatic
41	2	20.29	7	21	6.9	131000	HEB, SEC, PIMG	LB	no	unilateral SEC	normal	normal	asymptomatic
47	1	20.71	5.3	24.43	3.7	138000	normal	LB	no	normal	normal	normal	asymptomatic
51	2	24	6.4	25	4.9	166000	normal	LB	no	normal	normal	normal	asymptomatic
97	2	20.29	6.4	22.14	5.5	192000	normal	LB	no	normal	normal	normal	asymptomatic
1	1	27.43	5.04	29	4.3	156000	HEB, severe IUGR	LB	yes	normal	normal	normal	symptomatic
9	2	33.14	7.7	34.86	2.9	286000	HEB, SMG, LSC, SEC	LB	no	bilateral SEC, asymmetry of LV	normal	normal	symptomatic
11	2	32.86	7.5	35.71	3.1	199000	TC, HSWM, IVS, HMG, SMG	LB	no	mild VMG, bilateral SEC, bilateral LSC	normal	normal	symptomatic
13	1	21.86	7.2	22.86	5.7	196000	normal	LB	no	normal	normal	normal	symptomatic
33	1	24.86	7.7	32.57	3	254000	PIMG, HEB, LSC	LB	no	bilateral LSC	normal	normal	symptomatic
43	2	20.71	7.3	21.71	5.8	199000	HEB	LB	no	normal	bilateral	normal	symptomatic
45	2	27	7.3	28	5.5	110000	HEB, HSWM	LB	no	normal	bilateral	normal	symptomatic
49	1	23.14	8.1	24.86	4.3	109000	HSWM, HEB, SMG	LB	no	bilateral SEC, abnormal WM	bilateral	normal	symptomatic
55	1	23	8.4	25	5.7	55000	normal	LB	no	normal	bilateral	normal	symptomatic
57	2	19.86	6.8	20.57	4.7	231000	HEB	LB	no	normal	unilateral	normal	symptomatic
59	2	25	7.4	25.57	7	119000	normal	LB	no	normal	unilateral	normal	symptomatic
61	1	23.57	8.07	24.57	5.9	140000	HSWM	LB	no	normal	bilateral	normal	symptomatic
63	1	21.29	6	22.29	5.2	145000	normal	LB	no	bilateral LSC and SEC	bilateral	normal	symptomatic
53	2	34.57	7.4	36.71	5.9	NP	severe IUGR, severe MIC, HMG, SMG, SEC, LSC	TOP	NA	NA	NA	NA	Symptomatic and severe
65	NA	32	7.9	NA	NA	NP	mild bilateral VMG, PMG	TOP	NA	NA	NA	NA	Symptomatic and severe
67	1	22	6.95	NA	NA	NP	severe IUGR, severe MIC, PIMG, bilat VMG	ND	NA	NA	NA	NA	Symptomatic and severe
69	1	25.14	6.6	25.14	6.1	35000	bilateral VMG, LSC, hydrops, HMG	IUFD	NA	NA	NA	NA	Symptomatic and severe
71	2	17.14	6.7	21.14	3.6	11000	MIC, VMG, HEB, ASC, HMG, IUGR	TOP	NA	NA	NA	NA	Symptomatic and severe
73	2	22	6	26.86	5	NP	PMG, IVS, TC, HSWM, HMG, SMG	TOP	NA	NA	NA	NA	Symptomatic and severe
75	2	24.86	6.8	25.14	6.2	122000	IVS, TC, PMG	TOP	NA	NA	NA	NA	Symptomatic and severe
77	1	18	5.3	19.71	6.2	81000	MIC, VMG, HEB , PIMG, severe IUGR, ASC, PMG	TOP	NA	NA	NA	NA	Symptomatic and severe
79	2	24.71	7.1	26.71	4.4	119000	IUGR, HEB, HMG, HSWM, bilateral SEC and TC	TOP	NA	NA	NA	NA	Symptomatic and severe
81	1	17.86	6.3	NA	NA	NP	HEB, SEC	IUFD	NA	NA	NA	NA	Symptomatic and severe
83	2	21.29	5.8	23	4.8	100000	MIC, IEB	TOP	NA	NA	NA	NA	Symptomatic and severe
85	1	27.57	8.6	28.14	5.34	107000	HEB, MIC	TOP	NA	NA	NA	NA	Symptomatic and severe
87	NA	18.43	5	18.43	5	13000	HEB, ASC, hydrops, VMG	TOP	NA	NA	NA	NA	Symptomatic and severe
89	NA	20	NA	NA	NA	NP	Severe IUGR, MIC, LSC, cortical abnormalities	TOP	NA	NA	NA	NA	Symptomatic and severe
91	2	17.14	5.8	20.14	5	81000	IUGR, HEB, PMB, LSC	TOP	NA	NA	NA	NA	Symptomatic and severe
93	2	22	7.2	NA	NA	NP	????	TOP	NA	NA	NA	NA	Symptomatic and severe
95	1	23.57	6.5	25.14	4.3	129000	IUGR, VMG , IVS, PMG	TOP	NA	NA	NA	NA	Symptomatic and severe
99	2	27.29	7.7	29.71	5.5	NP	IUGR, SMG, HEB, MIC, VMG, LSC, HSWM	TOP	NA	NA	NA	NA	Symptomatic and severe

Table S2. Concentrations of all cytokines according to fetal in-fection, symptomatic status at birth, and severity (median and interquartile).

	Controls N=40	Infected fetuses N=40	Asymptomatic infected fetuses N=9	Symptomatic infected fetuses N=31	Symptomatic and non-severe infection N=13	Symptomatic and severe infection N=18	p-value infected vs. non-infected	p-value symptomatic vs. asymptomatic	Pre-processing filter
IL1a.interne	0 (0; 0)	0 (0; 0.0275)	0 (0; 0)	0 (0 ;0.1)	0 (0; 0)	0 (0; 0.3275)	0.4825	0.1624	
IL1b.interne	0.295 (0; 0.6)	0.35 (0; 1.225)	0 (0; 1.2)	0.4 (0 ;1.1)	0.4 (0.1; 1.3)	0.35 (0; 0.7)	0.2041	0.3107	x
IL2.interne	0 (0; 0)	0 (0; 0)	0 (0; 0)	0 (0 ;0)	0 (0; 0.49)	0 (0; 0)	0.3599	0.5403	
IL4.interne	0 (0; 0.925)	0 (0; 1.25)	0 (0; 0)	0 (0 ;1.4)	0.6 (0; 4.1)	0 (0; 0)	0.8363	0.9962	x
IL6.interne	0 (0; 0)	0 (0; 0.0099)	0 (0; 0)	0 (0 ;0.02)	0 (0; 0.19)	0 (0; 0.0099)	0.0566	**0.0110**	
IL8.interne	0 (0; 0)	0 (0; 0)	0 (0; 0)	0 (0 ;0)	0 (0; 0)	0 (0; 0)	0.3193	0.4666	
IL10.interne	0 (0; 0)	0 (0; 0)	0 (0; 0)	0 (0 ;0)	0 (0; 0)	0 (0; 0)	0.3868	0.4117	
IL13.interne	0 (0; 0)	0 (0; 0)	0 (0; 0)	0 (0 ;0)	0 (0; 0)	0 (0; 0)	0.3204	0.3748	
IL15.interne	3.275 (0; 8.785)	4.545 (0.8975; 8.425)	4.79 (3.22; 8.4)	4.14 (0.535 ;8.405)	5 (1.63; 9.94)	3.915 (0; 8.0475)	0.3575	0.2976	x
IL16.interne	0 (0; 18.575)	0 (0; 13.0525)	0 (0; 20.8)	0 (0 ;12.175)	0 (0; 9.72)	1.155 (0; 16.2325)	0.9409	0.4341	x
IL18.interne	0.3 (0; 0.97)	0.35 (0; 1.255)	0.25 (0; 0.79)	0.44 (0; 1.39)	0.6 (0.2; 1.02)	0.18 (0; 2.59)	0.2943	0.0672	x
IL33.interne	0 (0; 0)	0 (0; 0)	0 (0; 0)	0 (0 ;0)	0 (0; 0)	0 (0; 17.9325)	0.4690	0.2018	
CalgranulinA.interne	3.95 (0; 9.5875)	4.9 (0; 13.4875)	9.8 (2.9; 19.8)	4.8 (0 ;9.6)	5 (1; 9)	3.3 (0; 9.775)	0.3041	0.4902	x
CalgranulinC.interne	0 (0; 0)	0 (0; 0)	0 (0; 0)	0 (0 ;0.23)	0 (0; 0)	0 (0; 0.4817)	0.0941	0.0997	
Eotaxin.interne	0 (0; 0)	0 (0; 0)	0 (0; 0)	0 (0 ;0)	0 (0; 0)	0 (0; 0)	0.4777	0.7147	
GMCSF.interne	0 (0; 0)	0 (0; 0)	0 (0; 0)	0 (0 ;0)	0 (0; 0.1)	0 (0; 0)	0.6059	0.3303	
GroaCXCL1.interne	0 (0; 0.21)	0 (0; 6.7625)	0 (0; 0)	0 (0 ;8.165)	0 (0; 0)	0 (0; 12.6575)	0.3170	0.4657	x
HMGB1.interne	0 (0; 0)	0 (0; 0)	0 (0; 0)	0 (0 ;0)	0 (0; 0)	0 (0; 0)	NA	NA	
IFNg.interne	0 (0; 0)	0 (0; 0)	0 (0; 0)	0 (0 ;0)	0 (0; 0)	0 (0; 0)	0.5103	0.5126	
IP10.interne	135.945 (54.1875; 272.7225)	451.405 (79.335; 956.49)	118.04 (49.58; 605.312)	505.33 (84.27 ;1342.97)	717.53 (84.06; 1170.79)	315.52 (84.9775; 1419.735)	**0.0037**	**0.0006**	x
ITACCXCL11.interne	0 (0; 71.1825)	0 (0; 60.1925)	0 (0; 60.8)	0 (0 ;53.1)	0 (0; 59.99)	0 (0; 45.4725)	0.3464	0.3484	x
MCSF.interne	0 (0; 0)	0 (0; 0.6175)	0.05 (0; 0.1)	0 (0 ;0.635)	0.67 (0; 1.1)	0 (0; 0)	0.3038	0.4657	
MCP1.interne	0 (0; 2.39)	0.375 (0; 5.415)	0 (0; 2.57)	0.75 (0 ;5.52)	0.75 (0; 3.66)	2.13 (0; 9.1625)	0.0636	**0.0156**	x
MIG.interne	0 (0; 9.7825)	0.975 (0; 17.695)	0 (0; 0)	3.7 (0 ;18.655)	1.05 (0; 17.49)	10.05 (0; 18.8275)	0.1480	**0.0309**	x
MIP1a.interne	0 (0; 0.0325)	0 (0; 0.795)	0 (0; 0)	0 (0 ;0.87)	0 (0; 0.28)	0.305 (0; 1.1625)	0.3493	0.1648	x
MIP1b.interne	0 (0; 0)	0 (0; 0.5025)	0 (0; 0.86)	0 (0 ;0.44)	0 (0; 0)	0.1 (0; 0.455)	0.5587	0.2572	x
MIP3a.interne	0 (0; 0)	0 (0; 0)	0 (0; 1.9)	0 (0 ;0)	0 (0; 7.3)	0 (0; 0)	0.4146	0.9438	
RANTES.interne	0 (0; 1.8625)	1.65 (0; 5.65)	0 (0; 2.89)	2.08 (0 ;6.29)	0 (0; 1.58)	5.505 (1.81; 11.0875)	0.0585	**0.0046**	x
TGFb.interne	0 (0; 0)	0 (0; 0)	0 (0; 0)	0 (0 ;0)	0 (0; 0)	0 (0; 0)	0.5127	0.7202	
TNFa.interne	0 (0; 0)	0 (0; 0)	0 (0; 0)	0 (0 ;0)	0 (0; 0)	0 (0; 0)	0.5813	0.7608	
CRP.interne	0 (0; 58.8599)	0 (0; 236.895)	149.63 (0; 367.21)	0 (0 ;219.925)	210.31 (0; 283.93)	0 (0; 0)	0.3882	0.6479	x
TRAIL.interne	0 (0; 0)	0 (0; 0)	0 (0; 0)	0 (0 ;0)	0 (0; 0)	0 (0; 0)	0.6960	0.8590	
CXCL6.interne	0 (0; 0)	0 (0; 0)	0 (0; 0)	0 (0 ;0)	0 (0; 0)	0 (0; 0)	0.8545	0.7265	
CXCL13.interne	0 (0; 0)	0 (0; 0)	0 (0; 0)	0 (0 ;0)	0 (0; 0)	0 (0; 0)	0.1961	0.2485	
MIF.interne	70.14 (41.84525; 112.3987)	71.997 (43.4595; 123.2492)	97.789 (43.7904; 135.68)	70.762 (43.1595 ;105.6775)	95.667 (85.049; 154.972)	55.455 (40.0892; 71.9237)	0.3382	0.8890	x
IFNa.interne	0 (0; 0)	0 (0; 0)	0 (0; 0)	0 (0 ;0)	0 (0; 0)	0 (0; 0)	0.6177	0.2707	
IFNb.interne	0 (0; 0)	0 (0; 0)	0 (0; 0)	0 (0 ;0)	0 (0; 0)	0 (0; 0)	NA	NA	
IFNlambda.interne	0 (0; 0)	0 (0; 0)	0 (0; 0)	0 (0 ;0)	0 (0; 0)	0 (0; 0)	0.3204	0.3748	
IL1a.surface	0 (0; 0)	0 (0; 0)	0 (0; 0)	0 (0 ;0.25)	0 (0; 0.61)	0 (0; 0)	0.2782	0.2449	
IL1b.surface	0.6 (0.15; 1.6975)	0.7 (0; 1.725)	0.7 (0; 1.48)	0.7 (0 ;1.75)	1.2 (0.3; 1.8)	0.3 (0; 1.25)	0.4002	0.3632	x
IL2.surface	0 (0; 0.06)	0 (0; 0)	0 (0; 0)	0 (0 ;0)	0 (0; 1.71)	0 (0; 0)	0.5475	0.8693	
IL4.surface	0.65 (0; 2.075)	0 (0; 1.7)	0 (0; 2.2)	0 (0 ;1.45)	1 (0; 2.2)	0 (0; 0.375)	0.7242	0.3821	x
IL6.surface	1.095 (0.295; 2.5125)	1.51 (0.795; 2.3)	1.664 (0.8; 1.8)	1.5 (0.825 ;2.33)	1.11 (0.77; 2.64)	1.53 (0.92; 2.2525)	0.4305	0.1750	x
IL8.surface	10.57 (3.3375; 17.18)	15.375 (8.34; 25.2475)	15.96 (8.8; 18.57)	14.79 (8.04 ;25.495)	16 (9.4; 26.5)	14.685 (7.74; 22.8625)	0.2809	0.4180	x
IL10.surface	0 (0; 0.2)	0 (0; 0.725)	0.46 (0; 1)	0 (0 ;0.635)	0.57 (0; 0.86)	0 (0; 0.35)	0.7030	0.6180	x
IL13.surface	0 (0; 0)	0 (0; 0)	0 (0; 0)	0 (0 ;0)	0 (0; 0)	0 (0; 0)	0.2958	0.6105	
IL15.surface	2.875 (0; 6.03)	2.38 (0; 5.95)	1.85 (0; 2.704)	2.59 (0 ;6.2)	1.85 (0; 3.32)	3.925 (0; 9.25)	0.5612	0.1895	x
IL16.surface	6.5 (0; 44.2)	15.245 (0; 44.07)	14.5 (2.51; 81.53)	15.99 (0 ;37.35)	14 (0.92; 43.86)	17.555 (0; 29.29)	0.7923	0.6267	x
IL18.surface	0.865 (0.4225; 1.54)	1 (0.455; 1.3175)	1.03 (0.75; 1.2)	1 (0.44 ;1.565)	1 (0.47; 1.43)	0.915 (0.4275; 2.1875)	0.9936	0.9794	x
IL33.surface	0 (0; 0)	0 (0; 0)	0 (0; 0)	0 (0 ;0)	0 (0; 0)	0 (0; 0)	0.5053	0.8215	
CalgranulinA.surface	2.425 (0; 5.875)	2.15 (0; 6.0225)	6 (2.1; 6.8)	1.6 (0 ;4.1)	3.2 (1.6; 7.8)	0 (0; 2.125)	0.3121	0.4982	x
CalgranulinC.surface	0 (0; 2.465)	0.195 (0; 5.91075)	1 (0; 20.611)	0 (0 ;5.323)	0 (0; 11.708)	0 (0; 3.72525)	0.2983	0.4744	x
Eotaxin.surface	0 (0; 0)	0 (0; 0)	0 (0; 0)	0 (0 ;0)	0 (0; 0)	0 (0; 0)	0.7576	0.7457	
GMCSF.surface	0 (0; 0)	0 (0; 0.85)	0.6 (0; 0.8)	0 (0 ;0.9)	0.6 (0; 1.05)	0 (0; 0)	0.0694	0.6023	x
GroaCXCL1.surface	0.345 (0; 27.2825)	17.72 (0; 35.245)	31.87 (18.85; 41.08)	13.25 (0 ;24.945)	15.5 (9.99; 63.78)	10.675 (0; 23.975)	0.1099	0.3507	x
HMGB1.surface	0 (0; 0)	0 (0; 0)	0 (0; 0)	0 (0 ;0)	0 (0; 0)	0 (0; 0)	0.8278	0.5517	
IFNg.surface	0 (0; 0)	0 (0; 0)	0 (0; 0)	0 (0 ;0.6)	0 (0; 1.4)	0 (0; 0)	0.6026	0.7579	
IP10.surface	216.47 (71.6525; 393.6525)	775.625 (300.34; 1680.205)	264.38 (181.94; 704.448)	892.91 (587.435 ;2036.44)	740.69 (566.94; 892.91)	1374.01 (629.18; 3813.8825)	**0.0138**	**0.0005**	x
ITACCXCL11.surface	85.95 (16.6325; 144.5225)	94.45 (42.359; 233.42)	81.9 (56; 230.93)	96.1 (25.65 ;212.34)	96.1 (60.3; 249.09)	93.06 (12.825; 156.6975)	0.7501	0.9097	x
MCSF.surface	0 (0; 0.4)	0.2 (0; 1.315)	0.3 (0.2; 1.4)	0.2 (0 ;0.75)	0.7 (0; 1.3)	0.2 (0; 0.45)	0.3105	0.4710	x
MCP1.surface	9.365 (0; 19.95)	8.725 (3.2; 25.0475)	7.84 (3.89; 9.15)	10.34 (2.825 ;27.545)	5.91 (1.13; 32.6)	16.545 (5.3475; 26.1425)	0.8266	0.6142	x
MIG.surface	5.75 (0; 13.1075)	12.11 (4.0775; 28.7625)	4.67 (0; 19.7)	12.42 (5.15 ;35)	12.42 (5.51; 38.4)	13.785 (5.6675; 27.015)	0.2215	0.0982	x
MIP1a.surface	0.37 (0; 2.5)	0.25 (0; 2.4)	0.8 (0; 1.856)	0 (0 ;2.735)	0 (0; 3.01)	0.3 (0; 2.395)	0.6574	0.7688	x
MIP1b.surface	0.59 (0; 2.3825)	0.81 (0; 2.5075)	0.94 (0; 2.288)	0.8 (0 ;2.615)	2.1 (0; 3.23)	0.63 (0.1; 1.885)	0.3291	0.4797	x
MIP3a.surface	0 (0; 0)	0 (0; 0)	0 (0; 0)	0 (0 ;0)	0 (0; 27.93)	0 (0; 0)	0.2163	0.3943	
RANTES.surface	9.385 (4.02; 18.605)	16.165 (7.2425; 26.72)	16.6 (7.01; 25.22)	15.73 (8.41 ;27.44)	13.28 (7.01; 18.75)	23.15 (9.9525; 36.92)	0.4618	0.5717	x
TGFb.surface	0 (0; 0)	0 (0; 0.1)	0 (0; 2.7)	0 (0 ;0)	0 (0; 0.5)	0 (0; 0)	0.5096	0.2978	
TNFa.surface	0 (0; 0)	0 (0; 0)	0 (0; 0)	0 (0 ;0)	0 (0; 0.2)	0 (0; 0)	0.3007	0.9432	
CRP.surface	2993.57 (1885.88; 5824.6725)	3253.165 (2054.605; 6279.77)	2893.28 (1733.97; 3241.92)	3319.65 (2095.535 ;7397.78)	2121.79 (1816.45; 3264.41)	6068.58 (3347.9625; 14867.88)	0.1388	**0.0035**	x
TRAIL.surface	77.8392 (36.0268; 216.2042)	90.6635 (21.82125; 218.6225)	25.688 (7.6784; 101.64)	125.625 (27.66375 ;229.949)	89.695 (21.015; 133.802)	220.6135 (62.4187; 410.7662)	0.3280	**0.0302**	x

CXCL6.surface	3.0905 (0; 24.0825)	6.2535 (0; 18.9685)	7.403 (6.024; 17.118)	5.71 (0 ;19.166)	0 (0; 12.495)	6.507 (0; 21.9962)	0.2277	0.2115	x
CXCL13.surface	5.7545 (0.4927; 16.1917)	5.014 (2.99575; 19.50925)	4.8 (3.032; 9.604)	5.228 (3.4385 ;20.9185)	4.045 (2.887; 11.555)	7.505 (4.2272; 40.366)	0.2347	0.0984	x
MIF.surface	24.8035 (19.1287; 35.6095)	32.2985 (16.93525; 49.349)	52.643 (40.833; 60.085)	24.92 (14.2375 ;44.1495)	31.37 (20.6; 48.251)	19.454 (11.0162; 37.0332)	0.2872	0.6061	x
IFNa.surface	0 (0; 0)	0 (0; 0)	0 (0; 0)	0 (0 ;0)	0 (0; 0)	0 (0; 0)	0.5486	0.9789	
IFNb.surface	4.4765 (0; 13.777)	5.542 (0.2325; 22.14625)	18.043 (4.946; 22.744)	4.351 (0 ;18.7785)	15.61 (6.534; 32.629)	0.155 (0; 0.70775)	0.1249	0.8486	x
IFNlambda.surface	0 (0; 0)	0 (0; 0)	0 (0; 0)	0 (0 ;0)	0 (0; 0)	0 (0; 0)	0.3356	0.4606	
IL1a.soluble	2.592 (0; 8.844)	6.678 (1.536; 21.96)	3.48 (0.36; 6.516)	7.86 (2.322 ;34.674)	10.92 (2.4; 63.768)	5.538 (2.283; 24.12)	0.1818	0.2031	x
IL1b.soluble	2.274 (0.57; 3.081)	2.106 (0.96; 3.387)	1.236 (0.96; 3.84)	2.16 (0.96 ;3.252)	2.844 (1.752; 4.152)	1.962 (0.96; 2.526)	0.4703	0.7137	x
IL2.soluble	0 (0; 0.93)	0 (0; 1.644)	0 (0; 0.48)	0 (0 ;1.896)	1.392 (0; 3.24)	0 (0; 0)	0.5894	0.8662	x
IL4.soluble	4.14 (0; 9.06)	2.76 (0; 7.59)	2.4 (0; 10.32)	2.76 (0 ;7.2)	6.96 (0; 8.76)	2.28 (0; 4.59)	0.7798	0.7892	x
IL6.soluble	42.432 (20.535; 75.915)	83.424 (41.553; 145.986)	119.928 (42.348; 157.092)	83.292 (39.906 ;138.708)	96.564 (54.684; 135.648)	58.098 (23.286; 147.324)	0.4056	0.2763	x
IL8.soluble	403.842 (248.019; 828.822)	771.18 (387.147; 1121.802)	1043.796 (901.004; 1157.904)	554.148 (350.796 ;1112.064)	776.484 (498.48; 1118.628)	398.088 (177.504; 897.813)	0.7489	0.9703	x
IL10.soluble	0.96 (0; 2.16)	1.26 (0; 2.31)	1.2 (0; 2.4)	1.32 (0 ;2.22)	1.8 (1.2; 1.92)	1.26 (0; 2.37)	0.8463	0.6952	x
IL13.soluble	0 (0; 8.49)	0 (0; 5.445)	0 (0; 7.08)	0 (0 ;0)	0 (0; 23.64)	0 (0; 0)	0.4741	0.7128	x
IL15.soluble	47.766 (30.033; 86.601)	52.092 (44.058; 84.81)	46.644 (40.44; 53.868)	57.504 (45.702 ;96.144)	49.164 (44.988; 83.04)	69.648 (48.702; 97.647)	0.3239	0.1297	x
IL16.soluble	276.66 (128.862; 635.907)	264.024 (170.265; 443.604)	257.88 (173.256; 646.548)	270.168 (168.066 ;433.5)	358.92 (256.8; 480.552)	179.64 (149.307; 412.449)	0.7468	0.9240	x
IL18.soluble	4.206 (3.099; 7.05)	7.416 (4.29; 10.698)	6.48 (4.8; 9.24)	8.124 (4.26 ;11.562)	5.16 (4.2; 7.2)	10.308 (7.395; 11.85)	**0.0234**	**0.0060**	x
IL33.soluble	29.196 (0; 248.751)	0 (0; 200.427)	0 (0; 52.188)	0 (0 ;220.266)	0 (0; 0)	114.12 (0; 251.823)	0.4149	0.8534	x
CalgranulinA.soluble	24.9 (12.996; 47.25)	12.9 (9.09; 46.14)	11.76 (11.28; 25.56)	13.68 (8.46 ;59.46)	35.16 (8.52; 78.96)	11.46 (7.89; 53.199)	0.3060	0.4673	x
CalgranulinC.soluble	75.24 (37.506; 116.208)	54.5226 (30.2322; 105.816)	54.264 (30.5232; 247.2996)	54.7812 (25.3536 ;94.1226)	69.4032 (20.2128; 106.104)	48.1092 (31.1811; 76.0539)	0.3783	0.5952	x
Eotaxin.soluble	0 (0; 0)	0 (0; 0)	0 (0; 0)	0 (0 ;0)	0 (0; 298.32)	0 (0; 0)	0.8109	0.9108	
GMCSF.soluble	0 (0; 5.754)	3.3 (0; 8.454)	6.132 (2.82; 8.316)	0 (0 ;8.508)	6.6 (0; 11.664)	0 (0; 3.378)	0.2562	0.5263	x
GroaCXCL1.soluble	239.778 (163.338; 449.448)	300.192 (167.295; 681.591)	660.06 (368.352; 2956.212)	259.596 (127.224 ;453.066)	433.56 (313.836; 746.184)	164.85 (106.071; 250.467)	0.5483	0.6260	x
HMGB1.soluble	0 (0; 0)	0 (0; 0)	0 (0; 0)	0 (0 ;0)	0 (0; 0)	0 (0; 0)	0.8986	0.6355	
IFNg.soluble	0 (0; 0)	0 (0; 9.66)	0 (0; 0)	0 (0 ;10.26)	5.04 (0; 9.84)	0 (0; 9.045)	0.6023	0.7441	x
IP10.soluble	10852.986 (7354.974; 15979.596)	24155.274 (13671.278; 33171.693)	13415.432 (7824.636; 28769.988)	24195.876 (16469.694 ;36399.324)	27967.932 (18816.576; 39577.848)	24155.274 (15166.275; 28695.609)	**0.0000**	**0.0001**	x
ITACCXCL11.soluble	548.298 (384.462; 831.096)	838.956 (588.57; 1682.418)	822.924 (546.864; 1233.608)	1015.44 (609.666 ;1809.216)	1015.44 (628.452; 1651.2)	967.158 (601.602; 1825.788)	**0.0418**	0.0547	x
MCSF.soluble	1.56 (0; 6.639)	2.88 (0.996; 10.659)	3.36 (2.4; 17.28)	2.4 (0.918 ;7.812)	6 (4.92; 12.636)	1.242 (0.669; 3.39)	0.2978	0.4557	x
MCP1.soluble	657.264 (404.613; 1345.818)	1069.272 (710.067; 2085.813)	1472.644 (981.768; 2190.672)	973.164 (666.324 ;1938.594)	1135.836 (948.036; 1358.016)	739.41 (463.266; 2458.623)	0.1303	0.3403	x
MIG.soluble	384.33 (213.141; 518.274)	804.144 (567.321; 1407.342)	581.1 (480.096; 870.42)	833.58 (596.754 ;1564.182)	794.748 (601.728; 2640.036)	972.624 (470.157; 1407.978)	0.1183	0.0519	x
MIP1a.soluble	16.17 (9.12; 23.94)	14.64 (10.437; 22.632)	16.44 (11.64; 24.36)	14.4 (9.9 ;21.828)	15.12 (10.8; 24.36)	12.51 (9.87; 19.794)	0.3749	0.5665	x
MIP1b.soluble	26.766 (8.574; 58.05)	18.84 (10.335; 45.06)	44.64 (12.96; 90.12)	16.44 (10.23 ;30.846)	16.44 (12.36; 86.64)	16.02 (8.37; 26.139)	0.5326	0.8959	x
MIP3a.soluble	128.52 (97.08; 213.696)	159.906 (92.07; 272.025)	216.576 (152.784; 261.972)	130.62 (80.016 ;271.512)	184.692 (130.62; 310.464)	102.21 (64.848; 172.551)	0.3086	0.4941	x
RANTES.soluble	58.446 (32.901; 91.293)	79.614 (59.928; 131.268)	86.856 (79.764; 126.432)	71.292 (57.246 ;135.69)	70.812 (61.8; 101.112)	75.378 (51.87; 166.944)	0.3631	0.5440	x
TGFb.soluble	1.44 (0; 14.7)	0 (0; 13.143)	9 (8.16; 24.12)	0 (0 ;12.18)	0 (0; 22.56)	0 (0; 6.18)	0.8011	0.5309	x
TNFa.soluble	1.44 (0; 3.429)	1.224 (0.84; 2.619)	1.56 (1.2; 4.2)	1.2 (0.84 ;2.262)	1.2 (0.84; 3.6)	1.194 (0.87; 1.587)	0.3673	0.6383	x
CRP.soluble	725.232 (196.254; 1356.666)	757.836 (352.305; 2831.577)	646.2 (356.676; 772.752)	898.992 (374.388 ;2997.594)	699.528 (168.756; 898.992)	2841.414 (675.408; 7620.282)	0.0987	**0.0044**	x
TRAIL.soluble	180.4938 (98.5061; 346.6263)	299.2086 (141.0222; 802.9308)	174.888 (141.1776; 224.6172)	415.3464 (155.9106 ;888.8652)	245.2572 (140.556; 509.3052)	589.3542 (219.0777; 1095.1194)	**0.0123**	**0.0004**	x
CXCL6.soluble	20.2302 (0; 63.2763)	31.014 (0; 54.4239)	30.9048 (8.0184; 51.4464)	31.1232 (0 ;56.0778)	30.7788 (0; 34.6032)	34.4466 (0; 57.7317)	0.2920	0.4395	x
CXCL13.soluble	96.5466 (49.797; 123.0435)	92.1534 (47.1816; 162.3552)	102.7056 (61.9356; 170.3652)	90.1884 (43.3368 ;157.977)	82.26 (33.36; 149.4312)	95.5428 (64.5228; 187.8444)	0.2785	0.3149	x
MIF.soluble	485.5284 (322.758; 658.4703)	503.1714 (406.1358; 785.7092)	521.886 (486.2088; 600.8484)	494.0052 (370.6656 ;803.2602)	541.5804 (389.2356; 1335.8772)	472.695 (310.9098; 679.3071)	0.0963	0.3385	x
IFNa.soluble	0 (0; 0)	0 (0; 0)	0 (0; 0)	0 (0 ;0)	0 (0; 0)	0 (0; 0)	0.7555	0.5948	
IFNb.soluble	25.7334 (3.8958; 65.4315)	29.4576 (3.6126; 94.3128)	31.4472 (12.4596; 64.9956)	29.406 (2.6574 ;98.6832)	46.224 (15.1368; 150.6192)	21.396 (2.1384; 36.5508)	0.2712	0.8165	x
IFNlambda.soluble	3.45 (0; 109.911)	2.73 (0; 103.23)	0 (0; 21.89)	6.3 (0 ;136.491)	5.46 (0; 15.21)	106.089 (0; 147.0435)	0.3819	0.4653	x

Table S3. Prediction analysis based on principal component analysis. Contribution of each PC for infection and se-verity.

	Total variance explained	Infection			Severity		
		Beta	Se	p	Beta	Se	p
**PC1**	65.62%	8.8E-05	2.3E-05	0.00010	3.2E-05	7.7E-06	8.06E-05
**PC2**	22.73%	-3.5E-05	2.9E-05	0.23	-4.3E-05	1.4E-05	0.0019
**PC3**	4.98%	-5.6E-05	6.6E-05	0.40	-2.6E-05	3.0E-05	0.40
**PC4**	2.12%	-3.9E-05	8.0E-05	0.62	1.0E-05	4.7E-05	0.83
**PC5**	1.43%	3.3E-05	9.7E-05	0.73	9.4E-05	5.6E-05	0.10
**PC6**	1.09%	6.1E-06	1.1E-04	0.96	-4.7E-05	6.5E-05	0.47
**PC7**	0.52%	6.6E-05	1.6E-04	0.68	1.2E-04	9.4E-05	0.22
**PC8**	0.43%	-6.3E-05	1.8E-04	0.72	7.8E-06	1.0E-04	0.94
**PC9**	0.28%	3.4E-04	2.2E-04	0.13	2.5E-04	1.3E-04	0.050
**PC10**	0.22%	-1.7E-04	2.5E-04	0.49	-8.2E-05	1.5E-04	0.58
**PC11**	0.14%	4.2E-04	3.3E-04	0.21	2.7E-04	1.8E-04	0.14
**PC12**	0.11%	-5.0E-04	3.7E-04	0.17	-2.9E-04	2.0E-04	0.15
**PC13**	0.09%	-8.2E-06	3.9E-04	0.98	1.1E-04	2.3E-04	0.63
**PC14**	0.07%	-4.4E-04	4.6E-04	0.34	-5.8E-04	2.5E-04	0.022
**PC15**	0.05%	4.6E-04	5.6E-04	0.41	2.1E-04	2.9E-04	0.47
**PC16**	0.03%	-3.4E-04	6.4E-04	0.60	-2.7E-04	3.7E-04	0.46
**PC17**	0.03%	-1.1E-03	7.3E-04	0.12	-5.1E-04	3.8E-04	0.18
**PC18**	0.01%	1.1E-03	1.0E-03	0.29	3.4E-04	5.5E-04	0.54
**PC19**	0.01%	-7.4E-04	1.4E-03	0.59	-1.3E-04	8.1E-04	0.88
**PC20**	0.01%	1.2E-03	1.6E-03	0.45	1.4E-04	8.8E-04	0.88
**PC21**	0.00%	2.4E-03	1.7E-03	0.16	9.2E-04	9.6E-04	0.34
**PC22**	0.00%	-1.5E-03	1.9E-03	0.42	-1.0E-03	1.1E-03	0.34
**PC23**	0.00%	2.8E-03	2.4E-03	0.25	9.7E-04	1.4E-03	0.48
**PC24**	0.00%	-2.7E-03	2.6E-03	0.30	-2.5E-03	1.5E-03	0.10
**PC25**	0.00%	3.2E-03	2.8E-03	0.25	6.4E-04	1.6E-03	0.70
**PC26**	0.00%	-1.4E-03	3.3E-03	0.67	-8.3E-04	2.0E-03	0.67
**PC27**	0.00%	-3.2E-04	3.6E-03	0.93	-1.8E-04	2.2E-03	0.93
**PC28**	0.00%	-2.0E-02	8.0E-03	0.01	-5.4E-03	2.3E-03	0.021
**PC29**	0.00%	-1.2E-03	4.4E-03	0.79	-1.0E-03	2.6E-03	0.70
**PC30**	0.00%	-2.9E-03	4.8E-03	0.55	6.6E-04	2.9E-03	0.82
**PC31**	0.00%	1.7E-04	5.3E-03	0.97	1.1E-04	3.2E-03	0.97
**PC32**	0.00%	-5.8E-03	6.5E-03	0.37	-2.6E-03	3.8E-03	0.49
**PC33**	0.00%	4.1E-03	6.6E-03	0.53	3.8E-03	3.9E-03	0.33
**PC34**	0.00%	-2.8E-03	7.0E-03	0.68	-1.2E-03	4.1E-03	0.76
**PC35**	0.00%	-4.2E-03	7.5E-03	0.58	-2.7E-03	4.5E-03	0.55
**PC36**	0.00%	-6.3E-03	7.9E-03	0.43	-2.5E-03	4.6E-03	0.59
**PC37**	0.00%	1.4E-02	9.5E-03	0.13	7.4E-03	5.3E-03	0.17
**PC38**	0.00%	-8.6E-03	1.0E-02	0.39	-1.9E-03	5.9E-03	0.74
**PC39**	0.00%	1.0E-03	1.1E-02	0.93	1.6E-04	6.5E-03	0.98
**PC40**	0.00%	-5.4E-03	1.3E-02	0.68	-2.3E-03	7.7E-03	0.76
**PC41**	0.00%	9.4E-04	1.4E-02	0.95	-3.1E-03	8.4E-03	0.71
**PC42**	0.00%	-2.1E-02	1.6E-02	0.19	-1.6E-02	9.3E-03	0.09
**PC43**	0.00%	-5.4E-03	1.8E-02	0.76	-2.5E-03	1.1E-02	0.81
**PC44**	0.00%	1.1E-02	1.9E-02	0.55	1.1E-02	1.1E-02	0.31
**PC45**	0.00%	4.2E-02	2.2E-02	0.058	1.9E-02	1.2E-02	0.12
**PC46**	0.00%	2.4E-02	2.2E-02	0.28	9.6E-03	1.3E-02	0.46
**PC47**	0.00%	3.3E-02	2.4E-02	0.17	1.5E-02	1.4E-02	0.26
**PC48**	0.00%	2.7E-03	2.6E-02	0.92	1.2E-04	1.5E-02	0.99
**PC49**	0.00%	4.5E-02	2.9E-02	0.12	1.5E-02	1.7E-02	0.36
**PC50**	0.00%	2.6E-02	3.0E-02	0.39	3.0E-02	1.7E-02	0.08
**PC51**	0.00%	-2.5E-02	3.4E-02	0.45	-4.5E-03	2.0E-02	0.82
**PC52**	0.00%	-3.3E-02	3.7E-02	0.38	-1.6E-02	2.2E-02	0.46
**PC53**	0.00%	4.5E-02	4.0E-02	0.26	2.8E-02	2.3E-02	0.22
**PC54**	0.00%	1.3E-02	4.3E-02	0.77	1.4E-02	2.6E-02	0.59
**PC55**	0.00%	-6.1E-02	4.7E-02	0.19	-2.2E-02	2.6E-02	0.41

**PC56**	0.00%	-3.2E-02	5.0E-02	0.52	-1.6E-02	2.9E-02	0.59
**PC57**	0.00%	6.4E-02	6.0E-02	0.29	-6.6E-03	3.5E-02	0.85
**PC58**	0.00%	1.1E-01	6.7E-02	0.08	3.8E-02	3.7E-02	0.31
**PC59**	0.00%	-6.9E-02	6.8E-02	0.31	-1.4E-02	4.0E-02	0.73
**PC60**	0.00%	-5.0E-03	7.4E-02	0.95	2.0E-02	4.4E-02	0.65
**PC61**	0.00%	-1.2E-02	8.0E-02	0.88	2.0E-02	4.8E-02	0.67
**PC62**	0.00%	-1.5E-01	8.8E-02	0.08	-6.3E-02	5.0E-02	0.21
**PC63**	0.00%	3.5E-02	9.7E-02	0.71	2.0E-02	5.7E-02	0.73
**PC64**	0.00%	2.2E-01	1.2E-01	0.059	7.3E-02	6.6E-02	0.27
**PC65**	0.00%	5.2E-02	1.4E-01	0.70	4.1E-02	8.0E-02	0.61
**PC66**	0.00%	7.8E-02	1.5E-01	0.59	-1.4E-05	8.7E-02	1.00
**PC67**	0.00%	9.1E-02	1.8E-01	0.61	-1.9E-02	1.1E-01	0.86
**PC68**	0.00%	1.9E-02	2.0E-01	0.93	2.3E-02	1.2E-01	0.85
**PC69**	0.00%	-4.0E-03	2.3E-01	0.99	-2.4E-02	1.3E-01	0.86
**PC70**	0.00%	-1.9E-01	2.6E-01	0.46	-9.5E-02	1.5E-01	0.53
**PC71**	0.00%	-6.1E-02	2.8E-01	0.83	-3.0E-02	1.7E-01	0.86
**PC72**	0.00%	3.2E-02	3.1E-01	0.92	-1.9E-02	1.9E-01	0.92
**PC73**	0.00%	-2.5E-03	3.5E-01	0.99	6.4E-02	2.1E-01	0.76
**PC74**	0.00%	4.5E-01	4.1E-01	0.27	2.7E-01	2.4E-01	0.27
**PC75**	0.00%	1.8E-01	4.8E-01	0.71	1.0E-01	2.9E-01	0.73
**PC76**	0.00%	-1.6E-01	5.4E-01	0.76	-9.8E-02	3.2E-01	0.76
**PC77**	0.00%	6.5E-01	6.4E-01	0.31	3.3E-01	3.7E-01	0.37
**PC78**	0.00%	9.9E-01	7.4E-01	0.19	4.5E-01	4.3E-01	0.30
**PC79**	0.00%	6.4E-01	7.8E-01	0.41	2.2E-01	4.6E-01	0.64
**PC80**	0.00%	3.6E-01	9.2E-01	0.70	3.5E-01	5.4E-01	0.52
**PC81**	0.00%	5.2E-01	1.1E+00	0.63	-5.1E-01	6.4E-01	0.43
**PC82**	0.00%	-2.0E+00	1.7E+00	0.24	-1.2E+00	9.8E-01	0.21
**PC83**	0.00%	-5.1E-01	1.7E+00	0.77	-6.8E-02	1.0E+00	0.95

Figure S1. Pre-processing. (A) Distributions of all proteins (variance). (B) Proportion of non-zero values.

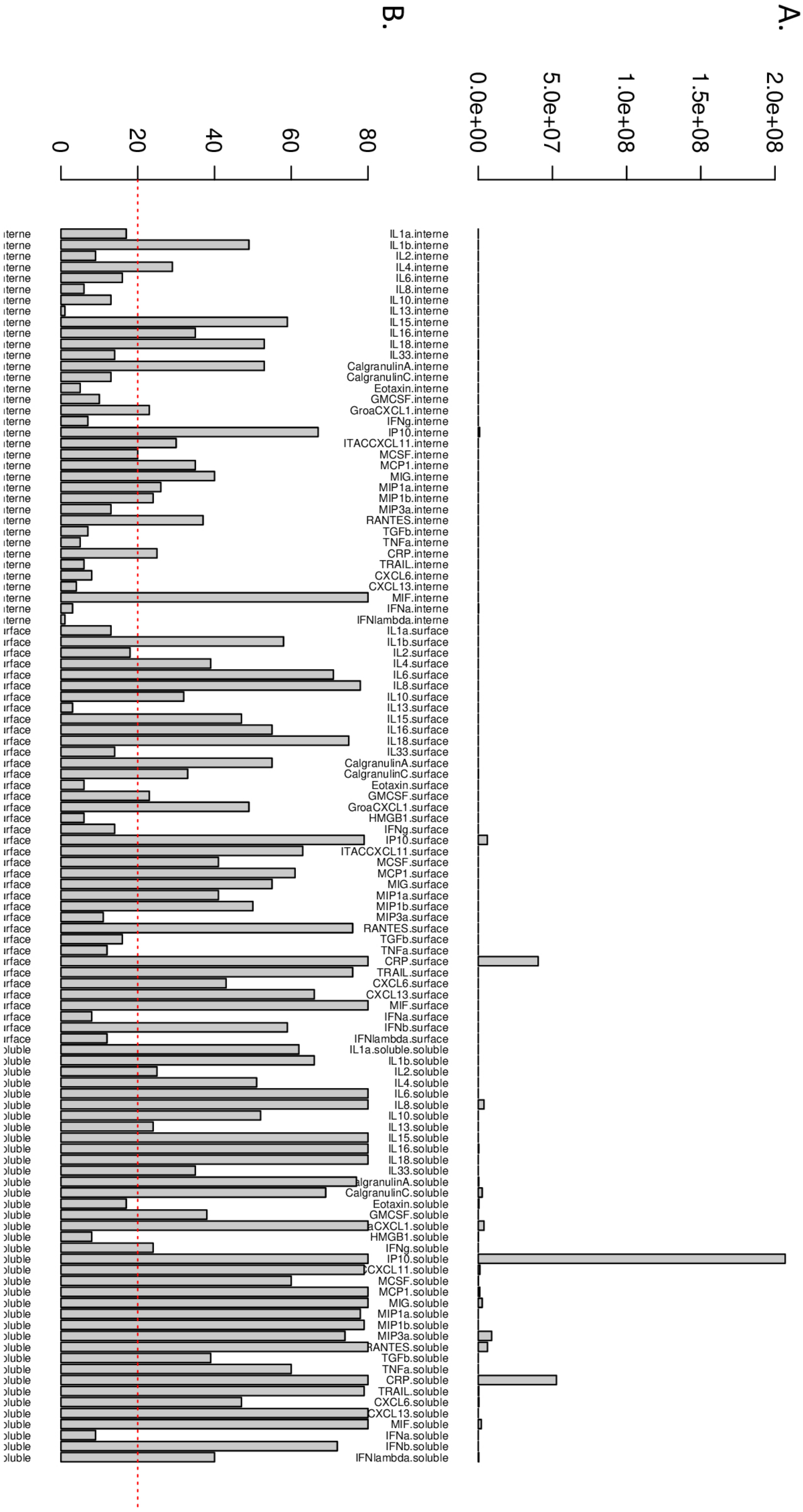

Figure S2. Data dimension reduction based on principal component analysis (PCA): cu-mulative variance explained by cellular classes are provided. (A) Internal cytokines. (B) Sur-face-linked cytokines. (C) Soluble cytokines. (D) Overall cytokines.

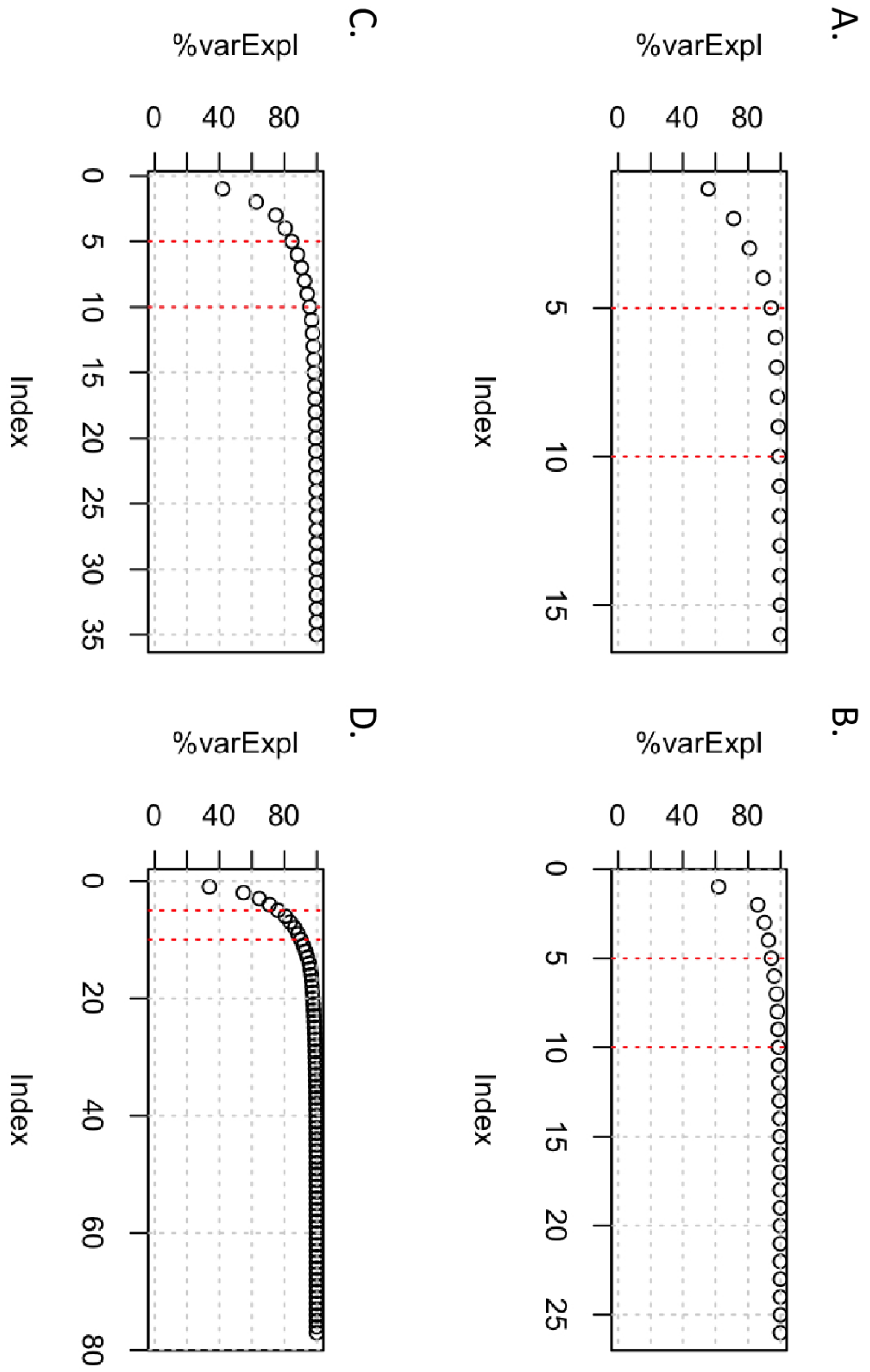

Supplement: Supplementary file 1 [file viruses-14-02145-s001.zip › viruses-1914497-Supplementary Materials.pdf]
